# Religion and health: most of times, an excellent combination

**DOI:** 10.31744/einstein_journal/2021CE6382

**Published:** 2021-02-25

**Authors:** Marcelo Saad, Roberta de Medeiros

**Affiliations:** 1 Associação Médico Espírita de São Paulo São PauloSP Brazil Associação Médico Espírita de São Paulo, São Paulo, SP, Brazil.; 2 Centro Universitário Lusíada SantosSP Brazil Centro Universitário Lusíada, Santos, SP, Brazil.

Dear Editor,

The letter of Pasternak^(1)^ raised controversial issues about the relationship between religion and health. We intend here to bring some elements for a conciliatory position. In the majority of research on the subject, religious-spiritual well-being is directly and positively associated with better parameters of physical and mental health, quality of life, and longevity.^(2)^ The benefit of religiosity to general health may be comparable to that are achieved from the consumption of fruits and vegetables.^(3)^

Thus, religiosity is not harmful to health. However, some religious interpretations may be inadequate, when selected parts of the scriptures are taken out of context. Sectarian or fanatical religious leaders can sometimes place the patient amid circumstances between medical and religious ethics. A dysfunctional belief system may encourage the patient to use the power of faith as a substitute for clinical care. A negative religious coping gives rise to spiritual distress and defensive behaviors, which affect treatment decisions, and well-being. Particular views about the sanctity of life may lead to dysthanasia, the persistence in futile treatments in terminal conditions.

On the other hand, a solid belief structure brings meaning, purpose, and connectivity. The result is a positive religious coping to face disease, manifested through strengthening, comfort, well-being, security, and idealism. To conclude, despite the constant challenge by issues raised by faith convictions, our role is to give the patients the best information and allow them to decide based on their values.^(4)^
