# Supplementary material for: Religion and health: most of times, an excellent combination
Source: Einstein (Sao Paulo). 2021 Feb 25;19:eCE6382. doi: 10.31744/einstein_journal/2021CE6382 (PMC7909003; doi:10.31744/einstein_journal/2021CE6382)
Supplement: Translation

# Translation: Religião e saúde: na maioria das vezes, uma excelente combinação

**Authors:** Marcelo Saad, Roberta de Medeiros

**Affiliations:** 1 Brazil Associação Médico Espírita de São Paulo, São Paulo, SP, Brazil.; 2 Brazil Centro Universitário Lusíada, Santos, SP, Brazil.

Caro Editor,

A carta ao editor de Pasternak^(1)^ levantou questões polêmicas sobre a relação entre religião e saúde. Nossa intenção é trazer alguns elementos para um posicionamento conciliatório. Na maioria dos estudos no assunto, bem-estar religioso e espiritualidade são diretamente e positivamente associados com melhores parâmetros de saúde física e mental, qualidade de vida e longevidade.^(2)^ O benefício da religiosidade para saúde em geral pode ser comparável aos benefícios alcançados a partir do consumo de frutas e vegetais.^(3)^

Contudo, a religiosidade não é prejudicial à saúde. Apesar disso, algumas interpretações religiosas podem, sim, ser nocivas, em particular quando partes das escrituras são posicionadas fora de contexto. Alguns líderes de seitas e fanáticos religiosos podem, em alguns momentos, colocar o paciente entre a medicina e a ética religiosa. Um sistema de crenças disfuncional pode incentivar o paciente a utilizar o poder da fé como substituto ao cuidado clínico. Um enfrentamento religioso negativo causa angústia espiritual e comportamento defensivo, o que afeta as decisões de tratamento e bem-estar. As visões particulares sobre santidade da vida podem levar à distanásia, isto é, a persistência em tratamento fúteis em condições terminais.

Por outro lado, uma estrutura de crenças sólida traz significado, objetivo e conectividade. O resultado é um enfrentamento religioso positivo para lidar com as doenças por meio do fortalecimento, do conforto, do bem-estar, da segurança e do idealismo. Com isso, apesar do desafio constante devido às questões levantadas por convicções de fé, nosso papel é o de disponibilizar aos pacientes a melhor informação e permitir que eles façam sua decisão baseada em seus valores.^(4)^
